# Interactive association of lipopolysaccharide and free fatty acid with the prevalence of type 2 diabetes: A community‐based cross‐sectional study

**DOI:** 10.1111/jdi.13056

**Published:** 2019-05-03

**Authors:** Xiuji Huang, Dan Yan, Mingtong Xu, Feng Li, Meng Ren, Jin Zhang, Muchao Wu

**Affiliations:** ^1^ Department of Endocrinology Sun Yat‐sen Memorial Hospital Sun Yat‐sen University Guangzhou China

**Keywords:** Free fatty acid, Lipopolysaccharide, Type 2 diabetes

## Abstract

**Aims/Introduction:**

Increased blood lipopolysaccharide (LPS) or free fatty acid (FFA) levels correlate with an increased risk of type 2 diabetes. The purpose of the present study was to evaluate the interactive effect of serum LPS and FFA levels on the prevalence of type 2 diabetes.

**Materials and Methods:**

This cross‐sectional study included 2,553 community‐dwelling Chinese adults. Fasting serum LPS levels were determined using the Limulus Amebocyte Lysate Chromogenic Endpoint assay, and FFA levels were determined using an enzymatic method. The participants were divided into three groups according to the tertiles of LPS or FFA levels or nine groups according to the tertiles of LPS and FFA levels. The odd ratios (ORs) for type 2 diabetes were estimated using logistic regression analysis.

**Results:**

We found that higher serum LPS or FFA levels were associated with higher high‐sensitivity C‐reactive protein levels (*P *<* *0.001), homeostatic model assessment of insulin resistance levels (*P *<* *0.001) and ORs for type 2 diabetes (*P *<* *0.01). Meanwhile, there were significant interactions between LPS and FFA in terms of the high‐sensitivity C‐reactive protein level (*P *<* *0.001), homeostatic model assessment of insulin resistance level (*P *<* *0.001) and ORs for type 2 diabetes (*P *<* *0.001). In the fully adjusted logistic regression model, the OR for participants with type 2 diabetes in the higher LPS and FFA level group were 6.58 (95% confidence interval 3.05–14.18, *P *<* *0.001) compared with that in participants in the lower LPS and FFA level group.

**Conclusions:**

The interaction between LPS and FFA was associated with an increased risk of type 2 diabetes in community‐dwelling Chinese adults.

## Introduction

Low‐grade inflammation in obesity is a risk factor for type 2 diabetes, and inflammation contributes to the pathogenesis of type 2 diabetes through the induction of insulin resistance (IR)^1^. Blood levels of lipopolysaccharide (LPS) or free fatty acid (FFA), an inducer of pro‐inflammatory cytokines, are related to the development of type 2 diabetes^2^, ^3^. However, whether the interaction between LPS and FFA is correlated with an increased risk of type 2 diabetes remains unknown.

Obesity is characterized by increased levels of circulating LPS and FFA^4^, ^5^. Circulating LPS are derived from Gram‐negative commensal bacteria in the intestinal tract, whereas FFA is released from adipose tissue^6^, ^7^. Aside from being an individual inducer of pro‐inflammatory cytokines^6^, ^7^, it was recently observed that LPS can promote the production of FFA, whereas FFA can enhance the pro‐inflammatory effects of LPS^8^, ^9^, indicating that there might be an interaction between LPS and FFA in inflammation.

Although LPS or FFA plays a significant role in the development of inflammation, treatment with an anti‐LPS antibody or antagonistic FFA receptor alone has had limited clinical success. For example, immunoglobulin G LPS, an antibody purified from hyperimmune bovine colostrum, has been shown to alleviate inflammation and IR in *ob*/*ob* mice^10^. However, the use of the LPS‐neutralizing antibody in humans did not result in a clear improvement in inflammation^11^. In contrast, lowering the levels of FFA with acipimox, an antilipolytic agent, lightly reduces IR in humans^5^, ^12^. However, GLPG0974, an antagonist of the FFA receptor 2 that has been shown to reduce inflammation *in vitro*, failed to benefit patients with chronic inflammatory diseases, such as ulcerative colitis^13^. If there are interactions between LPS and FFA on inflammation, IR, and the prevalence of type 2 diabetes, the treatment that decreases the production of both LPS and FFA or simultaneously blocks both effects of LPS and FFA would provide a more effective alternative for improving inflammation and IR, and thereby the treatment and/or prevention of type 2 diabetes.

Thus, we carried out the present study to investigate whether there was an interactive effect of serum LPS and FFA levels on systemic inflammation, IR, and type 2 diabetes in a community‐dwelling Chinese population.

## Methods

### Study population

The present cross‐sectional study was carried out from March to June 2015, in Guangzhou, China. The study population was from the Thyroid disorders, Iodine status, and Diabetes: A National Epidemiological Survey‐2014 (TIDE) study in China. The study was carried out in accordance with the Declaration of Helsinki, as revised in Fortaleza (October 2013), and was approved by the institutional review board of the Sun Yat‐sen Memorial Hospital of Sun Yat‐sen University with participants providing written informed consent. A total of 2,698 residents, aged 18–88 years, participated in the study, including 1,402 urban residents (living in Fangcun, Guangzhou) and 1,296 rural residents (living in Zengcheng, Guangzhou), respectively. Participants with a prescription for antidiabetic drugs (metformin, pioglitazone or insulin) or lipid‐regulating drugs (statins or fibrates; *n* = 81), which might decrease serum LPS or FFA levels^14^, ^15^, ^16^, ^17^; high‐sensitivity C‐reactive protein (hs‐CRP) levels ≥10 mg/L (*n* = 26), which indicates acute inflammations^18^; and missing hs‐CRP values (*n* = 38) were excluded from the study. Accordingly, 2,553 participants were included in the final data analyses.

### Diabetes definition

On the basis of the diagnostic criteria by the World Health Organization in 1999, diabetes is diagnosed as fasting plasma glucose (FPG) ≥7.0 mmol/L, 2‐h plasma glucose (2‐h PG) during an oral glucose tolerance test ≥11.1 mmol/L or self‐reported physician diagnosis of diabetes.

### Collection of clinical information

Information about age, sex, region, education history, drinking history, smoking history and medication, as well as history of diabetes and dyslipidemia was collected. The levels of physical activity at leisure time were estimated by the International Physical Activity Questionnaire. Body height and weight were recorded. Body mass index (BMI) was defined as weight in kilograms divided by the height in meters squared. Two measurements of blood pressure with at least a 10‐min interval were obtained using an automated electronic monitor (HEM‐8102A; Omron Healthcare, Co., Ltd., Tokyo, Japan), and the average of the two blood pressure measurements was used for analysis.

### Blood sample collection and measurements

After an overnight fast of at least 8 h, venous blood samples were collected. For participants without knowledge of their diabetic state, a 75‐g oral glucose tolerance test was then carried out, and blood samples were collected at 2 h after the administration of oral glucose.

For LPS and FFA, blood samples were centrifuged for 10 min; serum sample were obtained and stored at −80°C until use. Serum LPS was measured using the Limulus Amebocyte Lysate Chromogenic Endpoint assay (Hycult Biotec, Uden, the Netherlands); the intra‐assay and interassay coefficients of variability were 2.1% and 6.7%, respectively. Serum FFA was measured using the enzymatic method (Sekisui Medical Co., Ltd., Tokyo, Japan), and the intra‐assay and interassay coefficients of variability were 2.4 and 6.3%, respectively. The other blood biochemical markers, including FPG, fasting triglyceride (TG), low‐density lipoprotein‐cholesterol (LDL‐C), hemoglobin A1c, insulin, hs‐CRP and 2‐h PG levels, were measured as described in our previous study^19^.

Homeostatic model assessment of IR (HOMA‐IR) was calculated from glucose and insulin levels using the following equation: fasting serum insulin (mU/L) × FPG (mmol/L)/22.5.

### Statistical analysis

We used SPSS version 20 (SPSS Inc., Chicago, IL, USA) for statistical analyses. Continuous and normally distributed variables were presented as the mean ± standard deviation, continuous and non‐normally distributed variables were presented as a the median and interquartile range, whereas categorical variables were presented as percentages. The research participants were divided into three groups according to tertiles of serum LPS levels (lower tertile, <0.23 EU/mL; middle tertile, 0.23–0.52 EU/mL; and higher tertile, >0.52 EU/mL) or serum FFA levels (lower tertile, <442.92 μmol/L; middle tertile, 442.92–623.20 μmol/L; and higher tertile, >623.20 μmol/L). The hs‐CRP values were transformed to natural logarithms (Ln[hs‐CRP + 1]) and HOMA‐IR values were transformed to common logarithms (Log[HOMA‐IR]) before analysis because of the non‐normal distribution of these two variables, as described in previous studies^20^, ^21^. The trends across the LPS or FFA groups were analyzed using a simple linear regression analysis, or unadjusted and multivariate‐adjusted logistic regression analysis.

The interaction between serum LPS and FFA levels was analyzed by introducing the interaction term (serum LPS level multiplied by serum FFA level) in the models by the multivariate‐adjusted linear regression analysis for Ln(hs‐CRP + 1) or Log(HOMA‐IR), or unadjusted and multivariate‐adjusted logistic regression analysis for type 2 diabetes. Meanwhile, all participants were divided into nine groups according to tertiles of serum LPS and FFA levels. The Ln(hs‐CRP + 1) or Log(HOMA‐IR) values in these groups were analyzed using a simple linear regression analysis. Differences among groups were tested using one‐way analysis of variance (anova), and post hoc comparisons were carried out using Bonferroni correction. The prevalence of type 2 diabetes was assessed using the χ^2^‐test. Unadjusted and multivariate‐adjusted logistic regression analyses were used to estimate the prevalent risk of type 2 diabetes.

All statistical analyses were two‐sided, and *P *<* *0.05 was considered significant.

## Results

### Characteristics of the study participants by LPS or FFA levels

The mean age of the study participants was 45.64 ± 15.60 years, and 57.4% were women. The median serum LPS and FFA levels were 0.36 EU/mL (range 0.27–0.62 EU/mL) and 524.30 μmol/L (range 402.45–686.19 μmol/L), respectively. Among the 2,553 participants, 247 (9.7%) had type 2 diabetes. Tables 1 and 2 show the characteristics of the participants according to serum LPS and FFA levels (in tertiles), respectively. Individuals with higher serum LPS or FFA levels were older and had elevated levels of BMI, systolic blood pressure (SBP), FPG, 2‐h PG, hemoglobin A1c, TG, low‐density lipoprotein cholesterol (LDL‐C) and insulin, and included higher proportions of women and lower proportions of drinkers (*P *<* *0.05). Individuals with higher serum FFA levels, but not with higher serum LPS levels, had higher proportions of urban residents (*P *<* *0.05).

**Table 1:** Characteristics of the study population by lipopolysaccharide tertiles

Characteristic	Tertile of serum LPS (EU/mL)			*P* for trend
	Lower tertile <0.29 (*n* = 851)	Middle tertile 0.29–0.52 (*n* = 851)	Higher tertile >0.52 (*n* = 851)	
Age (years)	43.93 ± 15.05	44.62 ± 15.63	48.38 ± 15.76	<0.001
Female sex	526 (61.8%)	479 (56.3%)	460 (54.1%)	0.004
Urban residence	432 (50.8%)	450 (52.9%)	431 (50.6%)	0.584
Education				
Primary school or below	27 (3.2%)	48 (5.6%)	55 (6.5%)	0.009
Junior high school	181 (21.3%)	169 (19.9%)	199 (23.4%)	
Senior high school	374 (43.9%)	374 (43.9%)	374 (43.9%)	
Undergraduate school or above	269 (31.6%)	260 (30.6%)	223 (26.2%)	
Drinking	454 (53.3%)	415 (48.8%)	402 (47.2%)	0.032
Smoking	170 (20.0%)	178 (20.9%)	177 (20.8%)	0.879
Physical activity (h/week)				
<0.5	502 (59.0%)	503 (59.2%)	509 (59.9%)	0.732
0.5–1	215 (25.3%)	202 (23.8%)	193 (22.7%)	
≥1	134 (15.7%)	145 (17.1%)	148 (17.4%)	
BMI (kg/m^2^)	22.83 ± 3.38	23.36 ± 3.55	24.28 ± 3.90	<0.001
SBP (mmHg)	126.32 ± 19.22	129.62 ± 18.46	134.03 ± 20.16	<0.001
FPG (mmol/L)	5.10 ± 0.88	5.28 ± 1.05	5.62 ± 1.76	<0.001
2‐h PG (mmol/L)†	6.30 ± 2.17	6.68 ± 2.54	7.62 ± 3.79	<0.001
HbA1c (%)	5.52 ± 0.70	5.59 ± 0.82	5.81 ± 1.20	<0.001
TG (mmol/L)	1.28 ± 1.12	1.39 ± 1.10	1.72 ± 1.68	<0.001
LDL‐C (mmol/L)	3.09 ± 0.99	3.21 ± 0.96	3.39 ± 1.01	<0.001
Insulin (mU/L)	5.45 (4.18–7.28)	6.91 (5.21–8.53)	9.97 (7.63–12.82)	<0.001

Data are expressed as mean ± standard deviation for continuous and normally distributed variables, median with the interquartile range for continuous non‐normally distributed variables or numbers with percentages for categorical variables; *P* for trend across the groups was estimated using the simple linear regression analysis. ^†^A total of 59 participants without knowledge of their diabetes status did not undergo an oral glucose tolerance test, resulting in 2,494 participants that were analyzed. 2‐h PG, 2‐h plasma glucose post‐glucose loading during oral glucose tolerance test; BMI, body mass index; FPG, fasting plasma glucose; HbA1c, hemoglobin A1c; LDL‐C, low‐density lipoprotein cholesterol; LPS, lipopolysaccharide; SBP, systolic blood pressure; TG, triglyceride.

**Table 2:** Characteristics of the study population by tertiles of free fatty acid levels

Characteristic	Tertile of serum FFA (μmol/L)			*P* for trend
	Lower tertile <442.92 (*n* = 852)	Middle tertile 442.92–623.20 (*n* = 850)	Higher tertile >623.20 (*n* = 851)	
Age (years)	42.84 ± 14.50	45.72 ± 14.91	48.37 ± 16.82	<0.001
Female sex	508 (59.6%)	500 (58.8%)	457 (53.7%)	0.027
Urban residence	377 (44.2%)	425 (50.0%)	511 (60.0%)	<0.001
Education				
Primary school or below	24 (2.8%)	51 (6.0%)	55 (6.5%)	0.001
Junior high school	167 (19.6%)	181 (21.3%)	201 (23.6%)	
Senior high school	382 (44.8%)	390 (45.9%)	350 (41.1%)	
Undergraduate school or above	279 (32.7%)	228 (26.8%)	245 (28.8%)	
Drinking	452 (53.1%)	437 (51.4%)	382 (44.9%)	0.002
Smoking	173 (20.4%)	178 (21.0%)	174 (20.5%)	0.947
Physical activity (h/week)				
<0.5	512 (60.1%)	508 (59.8%)	494 (58.1%)	0.192
0.5–1	217 (25.5%)	190 (22.4%)	203 (23.9%)	
≥1	123 (14.4%)	151 (17.8%)	153 (18.0%)	
BMI (kg/m^2^)	22.98 ± 3.32	23.65 ± 3.73	23.83 ± 3.86	<0.001
SBP (mmHg)	125.38 ± 18.02	130.71 ± 19.56	133.89 ± 20.05	<0.001
FPG (mmol/L)	5.14 ± 1.04	5.33 ± 1.22	5.53 ± 1.58	<0.001
2‐h PG (mmol/L)†	6.10 ± 2.43	6.85 ± 2.69	7.67 ± 3.47	<0.001
HbA1c (%)	5.50 ± 0.73	5.63 ± 0.94	5.78 ± 1.09	<0.001
TG (mmol/L)	1.13 ± 0.76	1.38 ± 0.95	1.88 ± 1.90	<0.001
LDL‐C (mmol/L)	3.14 ± 0.99	3.27 ± 1.00	3.29 ± 0.98	0.042
Insulin (mU/L)	5.96 (4.31–7.96)	6.96 (5.10–9.04)	9.23 (6.66–11.79)	<0.001

Data are expressed as mean ± standard deviation for continuous and normally distributed variables, median with the interquartile range for continuous non‐normally distributed variables or numbers with percentages for categorical variables; *P* for trend across the groups was estimated using the simple linear regression analysis. ^†^A total of 59 participants without knowledge of their diabetes status did not undergo an oral glucose tolerance test, resulting in 2,494 participants analyzed. 2‐h PG, 2‐h plasma glucose post‐glucose loading during oral glucose tolerance test; BMI, body mass index; FFA, free fatty acid; FPG, fasting plasma glucose; HbA1c, hemoglobin A1c; LDL‐C, low‐density lipoprotein cholesterol; SBP, systolic blood pressure; TG, triglyceride.

### Association of serum LPS or FFA level with systemic inflammation, IR and type 2 diabetes

**Figure 1:**
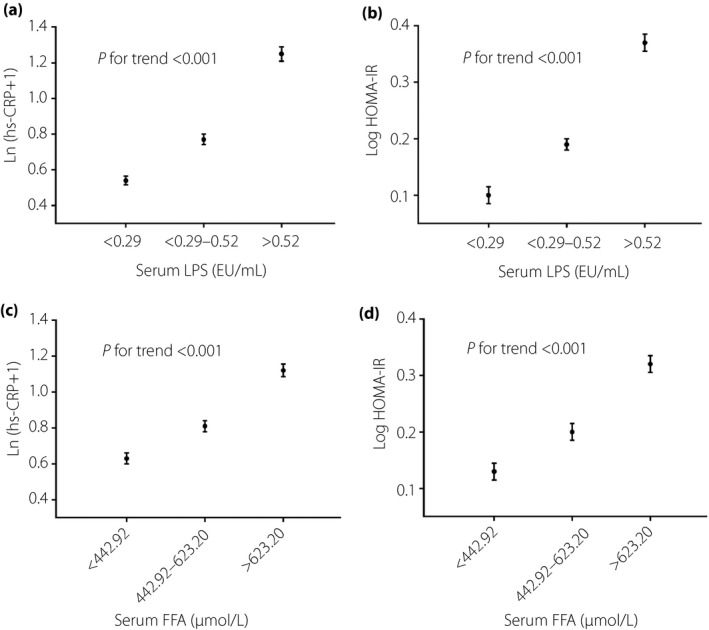
Association of serum lipopolysaccharide or free fatty acid (FFA) levels with systemic inflammation and insulin resistance. *P* for trend across the groups was estimated using the simple linear regression analysis. HOMA‐IR, homeostatic model assessment of insulin resistance; hs‐CRP, high‐sensitivity C‐reactive protein; LPS, lipopolysaccharide.

Figure 1 and Table 3 show the associations of LPS or FFA level with systemic inflammation, IR and type 2 diabetes. Increasing serum LPS or FFA levels were associated with higher Ln(hs‐CRP + 1) levels, Log(HOMA‐IR) levels and the prevalence of type 2 diabetes (*P *<* *0.001). There was a significant increasing trend of the odds ratios (ORs) for type 2 diabetes across increasing tertiles of LPS or FFA levels, even after adjusting for age, sex, region (urban/rural), education level, drinking, smoking, and levels of physical activity, BMI, SBP, TG and LDL‐C (*P *<* *0.01).

**Table 3:** Association of serum lipopolysaccharide or free fatty acid levels with the prevalence of type 2 diabetes

Tertile units	Prevalence of T2D (%)	OR (95% CI) for T2D	
		Unadjusted	Adjusted†
Serum LPS (EU/mL)			
Lower tertile (*n* = 851): <0.29	4.1	1	1
Middle tertile (*n* = 851): 0.29–0.52	8.5	2.16 (1.42–3.27)	2.05 (1.33–3.17)
Higher tertile (*n* = 851): >0.52	16.5	4.59 (3.13–6.74)	3.28 (2.18–4.93)
*P* for trend	<0.001	<0.001	<0.001
Serum FFA (μmol/L)			
Lower tertile (*n* = 852): <442.92	4.8	1	1
Middle tertile (*n* = 850): 442.92–623.20	8.8	1.91 (1.29–2.84)	1.41 (0.93–2.13)
Higher tertile (*n* = 851): >623.20	15.4	3.60 (2.50–5.18)	1.87 (1.25–2.78)
*P* for trend	<0.001	<0.001	0.007

*P* for trend across the groups was estimated using the simple linear regression analysis. ^†^Adjusted for age, sex, region (urban/rural), education level, drinking and smoking, and levels of physical activity, body mass index, systolic blood pressure, triglyceride and low‐density lipoprotein cholesterol. FFA, free fatty acid; LPS, lipopolysaccharide; T2D, type 2 diabetes.

### Interaction between serum LPS and FFA on systemic inflammation, IR, and prevalence of type 2 diabetes

**Figure 2:**
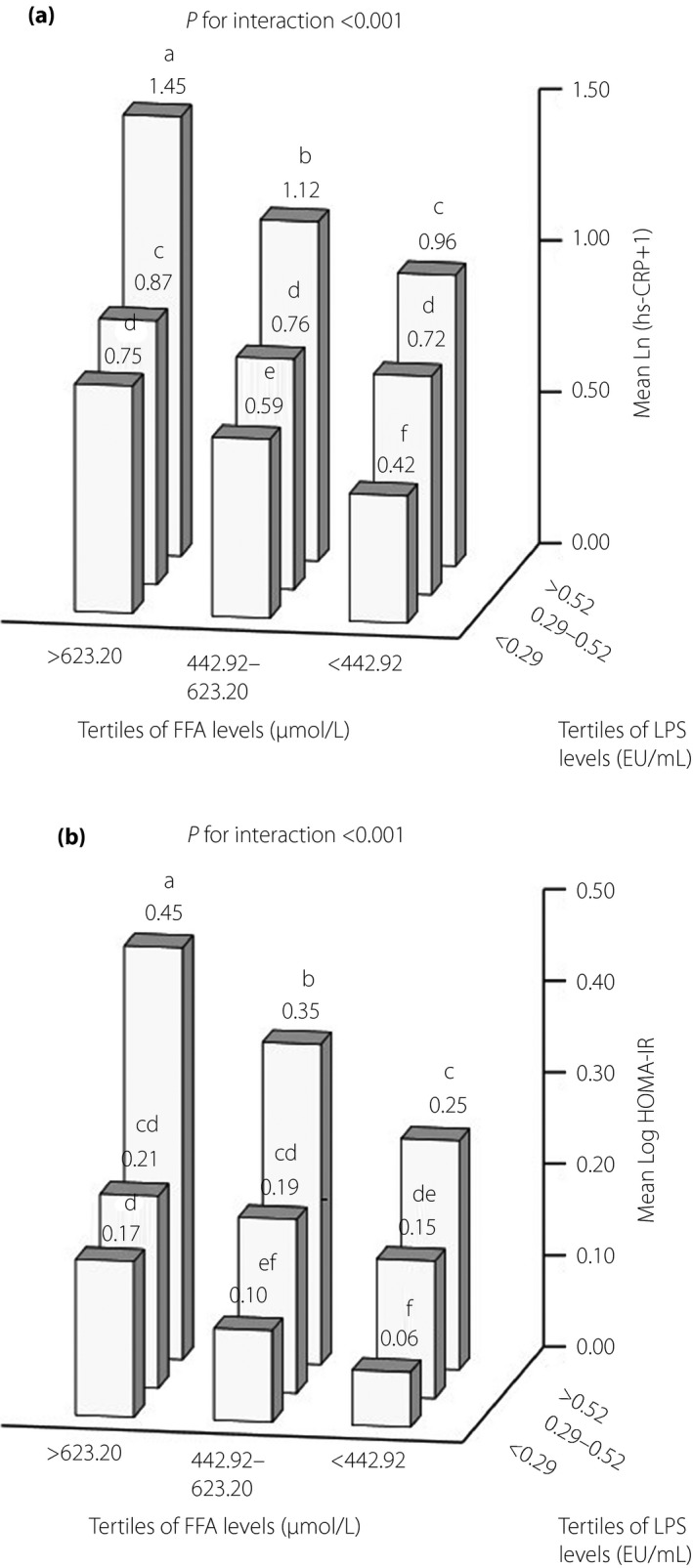
Interaction between serum lipopolysaccharide and free fatty acid (FFA) levels on inflammation and insulin resistance. The numbers above the bars indicate the values of (a) the mean high‐sensitivity C‐reactive protein (hs‐CRP) values transformed to natural logarithms (Ln[hs‐CRP + 1]) or (b) the mean homeostatic model assessment of insulin resistance (HOMA‐IR) values transformed to common logarithms (Log[HOMA‐IR]), and values not sharing a common letter above the numbers differ significantly at *P *<* *0.05. The effect of the interaction was estimated using linear regression analysis adjusted for age, sex, region (urban/rural), education level, drinking and smoking, and levels of physical activity, body mass index, systolic blood pressure, triglyceride and low‐density lipoprotein cholesterol. Differences among groups were tested using one‐way analysis of variance (anova), and post‐hoc comparisons were carried out using Bonferroni correction. LPS, lipopolysaccharide.

We found a significant interaction of LPS and FFA with Ln(hs‐CRP + 1) or log(HOMA‐IR) levels (both *P *<* *0.001) after adjusting for age, sex, region, education level, drinking, smoking, and levels of physical activity, BMI, SBP, TG and LDL‐C (Figures 2a,b). Participants in the higher LPS and FFA level group had higher levels of Ln(hs‐CRP + 1) (1.45, 95% CI 1.41–1.49 vs 0.42, 95% CI 0.40–0.44; *P *<* *0.001) or log(HOMA‐IR) (0.45, 95% CI 0.43–0.47 vs 0.06, 95% CI 0.05–0.08; *P *<* *0.001) than participants in the lower LPS and FFA level group (Figure 2a,b).

**Figure 3:**
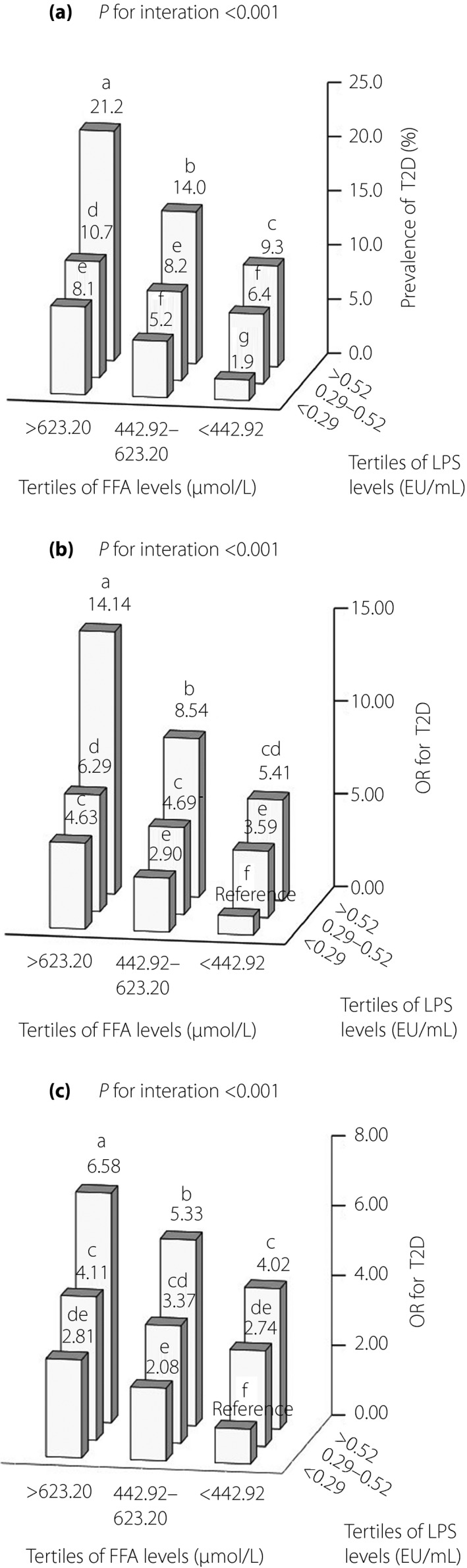
Interaction between serum lipopolysaccharide (LPS) and free fatty acid (FFA) levels on the prevalence of type 2 diabetes. The numbers above the bars indicate (a) the prevalence (%) of type 2 diabetes or (b,c) odds ratios (ORs) for type 2 diabetes, and values not sharing a common letter above the numbers differ significantly at *P *<* *0.05. The prevalence of type 2 diabetes was assessed using the χ^2^‐test. The ORs for type 2 diabetes were estimated by logistic regression analysis with participants with serum LPS <0.29 EU/mL and FFA <442.92 μmol/L as the reference group, and differences among groups were tested using logistic regression analysis. The effect of interaction was estimated using a logistic regression analysis. (b) The ORs for type 2 diabetes and the interaction were unadjusted. (c) The ORs for type 2 diabetes and the interaction were adjusted for age, sex, region (urban/rural), education level, drinking and smoking, and levels of physical activity, body mass index, systolic blood pressure, triglyceride and low‐density lipoprotein cholesterol. T2D, type 2 diabetes.

Similarly, there was a significant interaction between LPS and FFA levels in relation to the risk of type 2 diabetes, even adjusting for all covariates (*P *<* *0.001; Figure 3b,c). Participants in the higher LPS and FFA level group had a significantly higher prevalence of type 2 diabetes (21.1% vs 1.9%, *P *<* *0.001) than participants in the lower LPS and FFA level group (Figure 3a). In the logistic regression analysis, with the lower LPS and FFA level group as the reference group, the unadjusted and fully adjusted ORs for type 2 diabetes in participants in the higher LPS and FFA level group were 14.14 (95% CI 6.76–29.55, *P *<* *0.001) and 6.58 (95% CI 3.05–14.18, *P *<* *0.001), respectively (Figure 3b,c).

## Discussion

In the present study, we showed that LPS or FFA levels were related to systemic inflammation, IR and type 2 diabetes in a community‐dwelling Chinese population. Furthermore, we found, for the first time, a significantly interactive effect between LPS and FFA on systemic inflammation, IR, and type 2 diabetes. Participants with higher levels of both serum LPS and FFA tended to have higher levels of systemic inflammation and IR, and a higher prevalence of type 2 diabetes. This interactive effect on systemic inflammation, IR and prevalence of type 2 diabetes was independent of age, sex, region (urban/rural), education level, drinking and smoking, and levels of physical activity, BMI, SBP, TG and LDL‐C.

Previous studies have reported that blood LPS levels were increased in individuals with obesity or type 2 diabetes, and LPS has been associated with CRP, IR and the incidence of diabetes^2^, ^4^, ^22^. Similarly, here, we showed that higher serum LPS levels were also related to higher hs‐CRP levels (a well‐characterized, standardized biomarker of systemic inflammation), higher HOMA‐IR levels and higher prevalence of type 2 diabetes. LPS is an integral part of the outer membrane of Gram‐negative bacteria that are present in the gut microbiota^4^, ^23^. LPS can diffuse from the gastrointestinal tract into the bloodstream when intestinal permeability is increased^4^. Increased gut permeability has been observed in individuals with obesity^24^. Once in the systemic circulation, LPS binds to the Toll‐like receptor 4 present on the monocytes, macrophages and adipocytes, and then induces the expression and release of pro‐inflammatory cytokines, thereby resulting in IR^4^, ^25^, ^26^. The present results, combined with those from the aforementioned studies, showed that LPS plays a significant role in the development of inflammation, IR and type 2 diabetes.

Consistent with the other studies, including cross‐sectional and prospective studies^7^, ^27^, ^28^, ^29^, ^30^, ^31^, we showed that serum FFA levels were related to systemic inflammation, IR and prevalence of type 2 diabetes. FFA is used as the main source of fuel in the body during a fasting state. FFA is released into the circulation from adipose tissue and subsequently travels to reach its target tissues, such as the liver and skeletal muscle^7^. Blood FFA levels are increased in obese individuals, mainly because of increased FFA release, which has been associated with the expansion of fat mass^25^. Increased levels of FFA induced by an infusion of lipid in humans have been associated with IR^30^. FFA treatment *in vitro* induces inflammation and IR^26^, ^32^. FFA acts as an inducer of pro‐inflammatory cytokines and can activate Toll‐like receptor 4 to induce inflammation through the nuclear factor‐kappa B and c‐Jun N‐terminal protein kinase pathways; FFA also can activate G protein‐coupled receptor to promote inflammation through protein kinase C/nicotinamide adenine dinucleotide phosphate oxidase/reactive oxygen species/nuclear factor‐kappa B pathways^27^. Thus, FFA, at least partly through inflammation, is linked to the development of IR and type 2 diabetes.

Besides the separate effects of LPS and FFA on systemic inflammation, IR, and type 2 diabetes, we showed that the combination of elevated LPS or increased FFA level was related to increased systemic inflammation, IR and the prevalence of type 2 diabetes. There has been some evidence for the interaction of LPS and FFA in inflammation. As mentioned above, obese individuals, often with higher circulating FFA, have increased gut permeability^23^. A diet rich in saturated fatty acids is considered to elicit a metabolic inflammation by promoting intestinal permeation to LPS^33^. In contrast, injection of LPS causes a significant serum FFA increase *in vivo* 8. LPS has been shown to decrease the utilization of FFA by inhibiting triglyceride synthesis, suppressing the oxidation of FFA and stimulating lipolysis in adipose tissue, thereby increasing the circulating levels of FFA^8^, ^34^, ^35^. A low‐dose lipid infusion increases serum FFA levels, and monocytes exposed to lipid infusion *in vivo* enhance the *in vitro* secretion of pro‐inflammatory cytokines stimulated by LPS^5^. *In vitro* pre‐exposure to the saturated fatty acid palmitate can promote the pro‐inflammatory effect induced by LPS in microglia^36^. Recently, it was observed that there is a synergistic effect of LPS and palmitate on neuronal death in a Toll‐like receptor 4‐dependent manner^37^. Thus, apart from the individual pro‐inflammatory action of LPS and FFA, it is probable that both LPS and FFA can exert a synergistic effect on inflammation. Collectively, these results strongly support the concept that the interaction of LPS and FFA increases inflammation, thereby causing greater IR and higher prevalence of type 2 diabetes.

The mechanisms of *in vivo* chronic inflammation are very complex, and a single factor (such as LPS or FFA) alone might not fully account for the development of inflammation. Meanwhile, as already mentioned, treatment with anti‐LPS antibody or antagonist of the FFA receptor alone has had limited effects on inflammation in humans. Here, we showed that the interaction between LPS and FFA was related to increased systemic inflammation, IR, and the prevalence of type 2 diabetes. Therefore, decreasing the production of both LPS and FFA, or simultaneously blocking both effects, which synergistically attenuate inflammation, thereby further decrease IR in addition to their separate effects, and might be a more effective alternative for the treatment and/or prevention of type 2 diabetes.

The present study had some limitations. First, this study was based on a southern Chinese population, and thus the results from this study might not be representative of other populations. Second, information on diet was not collected in the present study; dietary patterns can have a potential effect on blood LPS or FFA levels^38^, ^39^. Finally, because of the cross‐sectional design of the study, no causal inference can be drawn from our results. Further investigation is required to clarify the effect of interactions between LPS and FFA on type 2 diabetes.

In summary, in the present study, we found that LPS or FFA levels were related to the presence of type 2 diabetes in a community‐dwelling Chinese population. More importantly, the interaction between LPS and FFA was associated with an increased risk of type 2 diabetes. Further investigation to validate these findings and show the mechanisms is necessary.

## Disclosure

The authors declare no conflict of interest.
